# Editorial: Family men: Fathers as coparents in diverse contexts and family structures

**DOI:** 10.3389/fpsyg.2022.975991

**Published:** 2022-07-22

**Authors:** Sarah E. DeMartini, Lauren E. Altenburger, Nancy L. Hazen, Martin I. Gallegos, Nicola Carone

**Affiliations:** ^1^Department of Psychology, California State University, Chico, CA, United States; ^2^Department of Human Development and Family Studies, Penn State Shenango, Sharon, PA, United States; ^3^Department of Human Development and Family Sciences, University of Texas at Austin, Austin, TX, United States; ^4^University of Texas at San Antonio, San Antonio, TX, United States; ^5^Department of Brain and Behavioral Sciences, University of Pavia, Pavia, Italy

**Keywords:** coparenting, fathers, gay fathers, diverse family contexts, underrepresentation

Fathers' involvement in childrearing has increased during the past several decades (Pattnaik, 2013; Livingston and Parker, 2019). Recent studies have suggested that fathers exert significant direct and indirect influences on child development and partner relationship quality *via* coparenting—how parents work with or against each other to care for their children (e.g., Farr and Patterson, 2013; Kuo et al., 2017; McHale et al., 2019; Schoppe-Sullivan and Fagan, 2020). High-quality father involvement has been shown to positively influence family dynamics (e.g., Green et al., 2019; DeMartini and Hazen, 2021). However, the majority of studies to date have focused largely on fathers' coparenting among middle class families headed by different-sex couples. Families across the globe have become increasingly diverse and children are parented within a variety of family structures. Thus, understanding the contribution of fathers across different contexts can offer new insights into modern fatherhood.

The goal of our Research Topic was to explore fathers' roles in coparenting with fathers as research participants. Nine contributing papers from across the globe examined fatherhood within same-sex couples, as well as in broader contexts that are more reflective of contemporary families and actual, representative familial experiences, such as fathers of multiple children, across family transitions, as well as from diverse cultural, ethnic, and racial backgrounds. Our Research Topic contributions highlight novel methods and analyses, which shed light on how fathers' involvement exerts unique influences on family dynamics.

The first seven articles in our Research Topic were quantitative research studies that explored the impact of fathers from diverse family structures on family members and dynamics. Notably, participating fathers come from across the globe, including China, Switzerland, Turkey, and the United States. Broad findings from the studies are summarized below.

Our first article explored the impact of psychological distress on parents' psychological flexibility (cognitive defusion, committed action, acceptance) on coparenting quality in Chinese fathers and mothers (Yu and Xiao). Structural equation modeling revealed that coparenting partially mediated the impact of anxiety on cognitive defusion and fully mediated the impact of depression on cognitive defusion and acceptance. Additionally, coparenting was found to moderate the associations between anxiety and cognitive defusion, as well as anxiety and acceptance.

The next article investigated the association between material hardship and children's prosocial behaviors by utilizing a risk and resilience framework in a socioeconomically disadvantaged sample of father-mother families of preschoolers in the United States (Lee et al.). Structural equation modeling revealed coparenting alliance related to higher levels of both mothers' and fathers' parenting, and in turn, responsive parenting was associated with higher levels of children's prosocial behaviors. Mothers' and fathers' responsive parenting mediated the indirect effects of coparenting alliance on children's prosocial behavior.

Results from Favez et al.'s study explored Swiss fathers' motivations to assume a particular role within the family across the transition to parenthood using multivariate general linear models. Findings from the study suggest reasons for role distributions were economical, practical, and to meet personal expectations. The coparenting relationship was shown to be impacted by age and deliberate choices in role distributions.

Our fourth article examined coparenting, parenting involvement, and children's school liking across the transition to primary school in China (Tau and Lau). Children's school liking was examined as a moderator between each parent's involvement in relation to coparenting cooperation and triangulation. Results highlighted the importance of paternal and maternal cooperation and the negative impact of triangulation on family dynamics.

Kara and Sümer's article showcased the importance of parental warmth and consistency on children's academic self-efficacy in Turkey *via* regression analyses. Findings suggested that fathers' warmth positively impacts boys' math self-efficacy. In addition, consistent coparenting efforts related to higher overall academic achievement.

Our sixth article explored the degree to which United States resident and non-resident fathers' coparenting as well as parenting quality and quantity related to children's ability to self-regulate their behaviors *via* path analyses (Altenburger). Findings from this study highlighted the importance of focusing on the promotion of positive father-child relationships in diverse family contexts. For instance, high levels of non-resident fathers' involvement related to children's increased ability to self-regulate.

Jacobvitz et al.'s study examined United States fathers' sensitivity and coparenting quality in the first 2 years of life following the transition to parenthood in relation to children's externalizing behavior in middle childhood. Structural equation modeling results suggested that fathers' caregiving quality plays an important role in determining coparenting quality and children's later externalizing problems.

The last two articles in our Research Topic article collection are opinion pieces, as informed by empirical work on same-sex fathers' coparenting and related topics. Broad suggestions made by the authors are summarized below.

Our first opinion article reviewed studies that explore the association between neurobiological activations and parental involvement by highlighting the opportunities and challenges of extending and conducting this research on male same-sex parents (Giannotti et al.). Authors suggested that despite difficulties in recruitment, collecting samples of diverse and arguably more representative family structures will provide valuable insight into our field.

Carone and Lingiardi's opinion article reviewed recent work that has explored gay fathers differentiation of caregiving and gender effects. Authors made three marked suggestions to researchers: (1) to consider caregiving roles and parent gender independently, (2) to make comparative analyses that assist in determining whether parent gender or adoption of complementary roles explains differences in coparenting behaviors, and (3) to acknowledge that caregiving roles vary based on contextual circumstances.

Broadly, our Research Topic suggests the importance of distinguishing between and disentangling gender from coparenting efforts, as well as the importance of more inclusive and representative research on fatherhood. Despite the contributions of our Research Topic to extant work on coparenting fathers, gaps remain in the literature. Taken together, we suggest that future researchers continue to examine both parents' perspectives of the coparenting relationship longitudinally, give further attention to same-sex fathers' coparenting in empirical studies, differentiate caregiving efforts from gender, and conduct more international work. Additionally, incorporating observations in naturalistic settings may prove useful in better understanding coparenting relationship quality across diverse family structures. It is our hope that readers will find this Research Topic to be a valuable reference and starting point from which to explore the role of contemporary fathers as coparents in diverse contexts.

## Author contributions

All authors listed have made a substantial, direct, and intellectual contribution to the work and approved it for publication.

## Conflict of interest

The authors declare that the research was conducted in the absence of any commercial or financial relationships that could be construed as a potential conflict of interest.

## Publisher's note

All claims expressed in this article are solely those of the authors and do not necessarily represent those of their affiliated organizations, or those of the publisher, the editors and the reviewers. Any product that may be evaluated in this article, or claim that may be made by its manufacturer, is not guaranteed or endorsed by the publisher.
